# A Formal Energy Consumption Analysis to Secure Cluster-Based WSN: A Case Study of Multi-Hop Clustering Algorithm Based on Spectral Classification Using Lightweight Blockchain

**DOI:** 10.3390/s22207730

**Published:** 2022-10-12

**Authors:** Yves Frédéric Ebobissé Djéné, Mohammed Sbai El Idrissi, Pierre-Martin Tardif, Ali Jorio, Brahim El Bhiri, Youssef Fakhri

**Affiliations:** 1 LRI, Faculty of Sciences, Ibn Tofail University, Kenitra 14000, Morocco; 2 SMARTiLab/EMSI, 49 Patrice Lumumba, Hassan, Rabat 10000, Morocco; 3 Department of Computer Science, Faculty of Sciences, Sherbrooke University, 2500 Bd de l’Université, Sherbrooke, QC J1K 2R1, Canada; 4 GRIC, Management School, Sherbrooke University, 2500 Bd de l’Université, Sherbrooke, QC J1K 2R1, Canada

**Keywords:** security, cryptography, clustering, energy, wireless sensors network

## Abstract

Wireless Sensors Networks are integrating human daily life at a fast rate. Applications cover a wide range of fields, including home security, agriculture, climate change, fire prevention, and so on and so forth. If WSN were initially flat networks, hierarchical, or cluster-based networks have been introduced in order to achieve a better performance in terms of energy efficiency, topology management, delay minimization, load balancing, routing techniques, etc. As cluster-based algorithms proved to be efficient in terms of energy balancing, security has been of less importance in the field. Data shared by nodes in a WSN can be very sensitive depending on the field of application. Therefore, it is important to ensure security at various levels of WSN. This paper proposes a formal modeling of the energy consumed to secure communications in a cluster-based WSN in general. The concept is implemented using the Proof of Authentication (POAh) paradigm of blockchain and applied to a Multi-hop Clustering Algorithm based on spectral classification. The studied metrics are residual energy in the network, the number of alive nodes, first and last dead node.

## 1. Introduction

WSN face many challenges: resource constraints in terms of energy and computational power, low transmission bandwidths and processing capabilities, and hidden terminals provoking collisions during data transmission [1,2]. Clustering techniques were proposed to tackle some of these issues. Compared with flat networks, cluster-based networks organize the network in smaller groups based on network characteristics and application requirements.

They logically divide WSN in units or clusters where different types of nodes are identified and assigned with roles:

- Cluster Members (CM): these nodes are attached to the same cluster. They read data from the physical environment.
- Cluster-Heads (CH): coordinate CMs, collect, aggregate, and forward data to a closer CH or to the sink.
- Gateways (optional): in some cluster-based WSN (CBWSN), gateways serve as relay point to forward data to other clusters or to maintain contact with close range clusters.
- Base Station (BS) or Sink: the final destination of data.

Ref. [2] defines clustering as a type of topology management techniques which can group nodes to improve the efficiency of the network by managing resources and rotating responsibilities among nodes to provide fairness. Key characteristics of CBWSN [1] include data fusion and load management, efficient energy management, relay node, collision avoidance, latency reduction, fault tolerance, deadlock prevention, quality of service (QOS), etc. In order to evaluate the performance of CBWSN, some elements to consider are:

- Cluster formation: the network model is either distributed or centralized. Cluster heads are selected using the node’s id, neighbor numbers, average hop distance, remaining energy, communication cost, and distance to the BS or to other cluster members. Clusters can be formed by grouping nodes and choosing CHs in these groups or by selecting first CHs and requesting CMs to connect to CHs. CHs are elected based on the availability of resources, randomly or in a predetermined way. CH election can be centralized or distributed.
- Cluster complexity: referred to the number of rounds required to perform the cluster formation and the transformation of data using broadcasting, multi-casting, or data aggregation
- Cluster communications and data forwarding: as nodes transfer data to CHs, it can be forwarded to the BS raw or combined with other information. Intra- and inter-cluster transmissions are executed using single hop or multi-hop routing depending on the position of the receiver and the distance between the sender and the endpoint.
- Cluster management: deals with network topology changes over time. Re-clustering may occur after a specific interval of time (time-based), or an event (event-based). For example, how is the topology affected when a a node enters, exits the network or runs out of energy?

According to [2], clustering algorithms have been developed to meet specific objectives, such as load balancing, scalability, packets delivery, throughput, delay, and 94.8% of surveyed articles in the CA field listed energy consumption as their objective. These clustering techniques were implemented using clear communications and did not integrate security features, such as cryptography. This study formally evaluates the costs generated by the addition of security elements in cluster-based WSN. The concept is implemented using PoAh, a lightweight Blockchain paradigm, applied to a Multi-hop Clustering Algorithm based on spectral classification. The paper is organized as follow: Section 2 examines clustering algorithms and security features. Section 3 covers Multi-hop Clustering Algorithm based on spectral classification and formal modeling of costs generated to secure communications, as well as the simulation environment. Section 4 presents results and interpretations with regards to residual energy, the number of alive nodes, first, half, and last dead nodes.

## 2. Related Work

Research in clustering algorithms for WSN has been prolific [3]. LEACH [4], EEHC [5], HEED [6], SEDEEC [7], ECFP [8], EAFCA [9], EERC [10], and EACLE [11] are examples of clustering algorithms used to enhance performances in WSN. These clustering techniques mainly focus on “open” clustering that is without security features.

Current applications require modern and powerful security techniques because they handle sensitive data depending on the field of application. Several propositions emerged to address security in cluster-based WSN.

An Energy Aware Trust-based Secured Routing Algorithm (EATSRA) [12] was proposed to evaluate the trust reputation of different nodes in the network. The end goal was to create a secure path to reach the BS using trust metrics such as trust scores and node behavior in order to identify safe paths.

Ref. [13] focused on CH election based on trust with the objective of detecting malicious cluster heads. Ref. [14] proposed a lightweight trust-based secure clustering in WSN using honeybee mating intelligence approach, while [15] used blockchain technology in WSN.

Previous works [16,17] introduced Proof-of-Authentication (PoAh) paradigm in blockchain which ensures integrity, confidentiality, and authentication with less requirements in terms of CPU usage. Ref. [18] adapted it to secure a flat and centralized network. Although these techniques address security with regards to various attacks, most of them do not evaluate the impact of security on energy consumption. The objective of this paper is to secure a clustering algorithm in WSN and evaluate the induced cost in terms of energy. This paper implements a secure Multi-hop Clustering Algorithm based on spectral classification (MHCA-SC) [19] using PoAh.

## 3. Methodology

The Multi-hop Clustering Algorithm based on spectral classification steps are summarized in the following subsections:

### 3.1. Pre-Processing Phase

**Figure 1:**
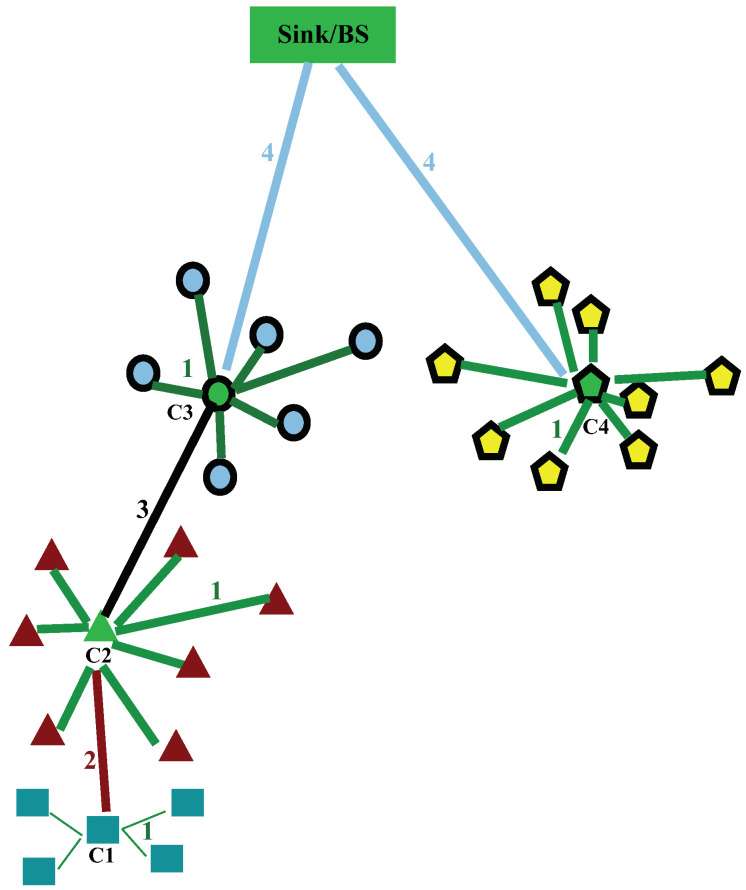
Communication model in MHCA-SC.

Using the RSS (Received Signal Strength), each node calculates its distance to the BS in order to define the region it belongs to (Figure 1). The information is conveyed to the BS which builds a graph and determines a similarity matrix A based on the following equation:

(1) $$A=\left[a_{ij}\right]=\left\{\begin{array}{cc} exp(\frac{-d^{2}\left(i,j\right)}{2\sigma^{2}}), & \mathrm{if}\;i\;\neq \;j\\ 0, & \mathrm{otherwise} \end{array}\right.$$

where d(i,j) is the euclidean distance between *i* and *j* and σ is the standard deviation or the mean distance of a point to its nearest neighbors.

The degree and Laplacian matrices are then generated.

### 3.2. Clustering Phase

Based on K-means classification and the Laplacian matrix, the main goal of this step is to determine the optimal number of clusters and assign each node to its corresponding cluster.

### 3.3. Cluster Head Election Phase

CHs are then determined and selected based on nodes’ id and their remaining energy in each cluster. For a node to be elected as a CH, its residual energy must be greater than the mean residual energy in the cluster, while the node’s id equals to Ck = (r mod|Sk|) where r is the round and Sk the number of nodes in the cluster. All nodes with a remaining power lower than Ermin notify node Ck + 1 of its energy.

### 3.4. Intra-Cluster Routing

In the same cluster, nodes communicate directly with their CH using schedule TDMA (Time Division Multiple Access).

### 3.5. Inter-Cluster Multi-Hop Routing

Although nodes easily reach their CH using single hop communications in the same cluster, packets are sent from CHs to the sink using a multi-hop routing technique (Figure 1).

### 3.6. Securing MHCA-SC

Compared with HCA-SC [20], SCNOC [21], DECSA [22], and LEACH-C [23], MHCA-SC proved to be more efficient, considering the FDN (first dead node) and the total residual energy in the network. However, with the growing number of attacks on WSN and their consequences, it is important to introduce security techniques in order to protect the network. In [18], a lightweight blockchain for secured WSN in single hop and centralized wireless networks was implemented. Prior to sending a message to the sink, each node hashes its MAC address and the message to send using SHA256 or MD5. Mac and message hashes are merged, then encrypted using AES256 and sent to the sink as detailed in Algorithm 1. The sink decrypts data (hashes) and authenticates the node by comparing the received values with a list of trusted nodes details, as described in Algorithm 2.
**Algorithm 1** Node**Require:** MACAddress**Ensure:** Send Message to the sink nodeMACadd←readContikiMAC(); textData←“Hello”; hashMAC←hash(nodeMACadd); **while** true **do**  startTimer(timer);  hashText←hash(textData);  dataSend←merge(hashMAC,hashText);  **if** timer is expired **then**    send(dataS,sinkId);  **end if** **end while**

**Algorithm 2** Sink
**Require:** node (nodeId, MACAddress, hashMAC)**Require:** TrustedNodes: Array of nodes**Ensure:** Accept or reject a message initTrustedNodes(); **while** true **do**  **if** messageReceived== true **then**    [hashMAC,hashText,nodeId]←extract(datas);    r←check(nodeId,hashMAC);    **if** *r*==1 **then**      $print{(}^{\prime }Trustednode:Accept^{\prime })$;    **else if** *r* ==2 **then**      $print{(}^{\prime }MaliciousNode:Reject^{\prime })$;    **else if** *r* ==0 **then**      $print{(}^{\prime }Unknownnode:Reject^{\prime })$;    **end if**  **end if** **end while**


The main challenge of securing clustering algorithms in WSN and specifically MHCA-SC, is to identify where cryptography takes place in the communication model. Messages in clustering in general and MHCA-SC are exchanged from (Figure 1):

- Node to CH (intra cluster): Each node within a cluster sends data to its CH (1 in Figure 1);
- CH to CHs (inter cluster): CHs cannot directly reach the sink and forward their data to CHs that relay to other CHs (2 in Figure 1) or to CHs that can easily reach the BS (3 in Figure 1);
- CH to BS/sink: these CHs directly access the sink with single hop communications (4 in Figure 1).

Several scenarios are described below to secure MHCA-SC.

1. Securing Node to CH communications: each node computes hashes, encrypts the data and sends it to the CH. Here, the added energy is the energy required to hash the message and the MAC address and finally cypher the combined data. Extra energy is calculated as follows:
  (2) $$\;E_{NCH}=\sum_{n=1}^{t}E_{nch}=\sum_{n=1}^{t}\left(2\cdot E_{hash}+E_{enc}\right)$$
  where $E_{nch}$ is the energy spent to secure communications between a node and its CH, $E_{hash}$ the energy spent to compute a hash, $E_{enc}$ the energy used to encrypt, *t* is the number of nodes in the cluster.
2. Securing CH to CHs (inter cluster) communications: The choices available in this scenario are:
  (a) A CH deciphers messages received from every single node within its cluster in order to authenticate the sender. Upon a successful authentication, the CH aggregates hashes, encrypts and forwards data to the next hop (steps b or c depending on the position of the CH and the cluster). Added energy in this case is calculated as follows:
    (3) $$\;E_{CH1}=\sum_{n=1}^{t}\left(E_{dec}+E_{auth}\right)+\left(2\cdot E_{hash}+E_{enc}\right)$$
    where $E_{CH1}$ is the energy spent to secure communications between a CH and another CH, $E_{dec}$ the energy spent to decrypt each message, $E_{auth}$ the energy spent to authenticate a node.
3. CH aggregates data received from nodes within its cluster (already encrypted by nodes) and forwards it to the next hop (steps b or c). Here, the added energy is
  (4) $$\;E_{CH1}=0$$
4. If a CH receives data from another CH, it either decrypts the data, authenticates the sender (another CH) and relays it (encryption) with the resulting equation:
  (5) $$\;E_{CH2}=\left(E_{dec}+E_{auth}\right)+\left(2\cdot E_{hash}+E_{enc}\right)$$
5. Or directly forwards it to the next CH.
  (6) $$\;E_{CH2}=0$$
6. Securing CH to BS communications: CHs are directly in contact with the Base Station/sink.
  (a) CH deciphers messages received from other CHs. If the sender (CH) is authenticated, data are prepared and securely sent to the sink. The resulting energy is:
    (7) $$\;E_{CHS}=\left(E_{dec}+E_{auth}\right)+\left(2\cdot E_{hash}+E_{enc}\right)$$
    where $E_{CHS}$ is the energy spent to secure communications between a CH and the sink.
  (b) Otherwise, the CH conveys data to the sink without any processing.
    (8) $$\;E_{CHS}=0$$

Table 1 highlights the required amount of energy to send messages from cluster1 to the base station based on Figure 1 in various configurations ($E_{1–8}$). The overall consumption of energy in the network includes energy costs generated by clusters 2, 3, and 4.

Where:

(9) $$\begin{array}{cc} E_{1} & =\sum_{n=1}^{t}\left(2\cdot E_{hash}+E_{enc}\right)+\sum_{n=1}^{t}\left(E_{dec}+E_{auth}\right)+\left(2\cdot E_{hash}+E_{enc}\right)\\ & +\left(E_{dec}+E_{auth}\right)+\left(2\cdot E_{hash}+E_{enc}\right)+\left(E_{dec}+E_{auth}\right)+\left(2\cdot E_{hash}+E_{enc}\right) \end{array}$$

(10) $$\begin{array}{cc} E_{2} & =\sum_{n=1}^{t}\left(2\cdot E_{hash}+E_{enc}\right)+\sum_{n=1}^{t}\left(E_{dec}+E_{auth}\right)+\left(2\cdot E_{hash}+E_{enc}\right)\\ & +\left(E_{dec}+E_{auth}\right)+\left(2\cdot E_{hash}+E_{enc}\right) \end{array}$$

(11) $$\begin{array}{cc} E_{3} & =\sum_{n=1}^{t}\left(2\cdot E_{hash}+E_{enc}\right)+\sum_{n=1}^{t}\left(E_{dec}+E_{auth}\right)+\left(2\cdot E_{hash}+E_{enc}\right)\\ & +\left(E_{dec}+E_{auth}\right)+\left(2\cdot E_{hash}+E_{enc}\right) \end{array}$$

(12) $$\begin{array}{cc} E_{4} & =\sum_{n=1}^{t}\left(2\cdot E_{hash}+E_{enc}\right)+\sum_{n=1}^{t}\left(E_{dec}+E_{auth}\right)+\left(2\cdot E_{hash}+E_{enc}\right) \end{array}$$

(13) $$\begin{array}{cc} E_{5} & =\sum_{n=1}^{t}\left(2\cdot E_{hash}+E_{enc}\right)+\left(E_{dec}+E_{auth}\right)+\left(2\cdot E_{hash}+E_{enc}\right)\\ & +\left(E_{dec}+E_{auth}\right)+\left(2\cdot E_{hash}+E_{enc}\right) \end{array}$$

(14) $$\begin{array}{cc} E_{6} & =\sum_{n=1}^{t}\left(2\cdot E_{hash}+E_{enc}\right)+\left(E_{dec}+E_{auth}\right)+\left(2\cdot E_{hash}+E_{enc}\right) \end{array}$$

(15) $$\begin{array}{cc} E_{7} & =\sum_{n=1}^{t}\left(2\cdot E_{hash}+E_{enc}\right)+\left(E_{dec}+E_{auth}\right)+\left(2\cdot E_{hash}+E_{enc}\right) \end{array}$$

(16) $$\begin{array}{cc} E_{8} & =\sum_{n=1}^{t}\left(2\cdot E_{hash}+E_{enc}\right) \end{array}$$

**Figure 2:**
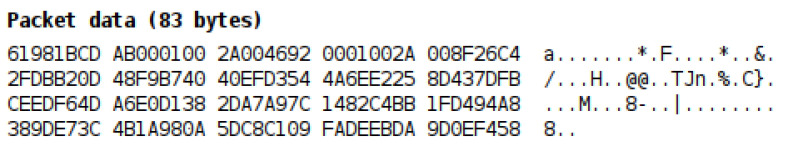
Sample Packet.

It is clearly noticeable that hashing and encrypting messages at every stage of the transmission increases the energy consumption in the network. In order to ensure minimal energy consumption, path $E_{8}$ is the best choice. In this case, hashes are computed and then encrypted by the node, while cluster heads only forward the encrypted data to the base station. The sink deciphers and authenticates each received message. Figure 2 illustrates a packet sent by a node to its CH and forwarded to the base station combined with packets from the same cluster. Encryption provides confidentiality while hashing ensures integrity: any data alteration by a malicious node during transportation is detected by the sink because the sink maintains a list of trusted nodes. Any node spoofing a trusted node MAC is considered as malicious by the sink because the node’s ID, MAC address, and the corresponding hashes will not match. It is also important to note that the sink is considered as a device with unlimited power and CPU resources compared with other nodes. As a result, it manages the majority of the computation load in the network.

### 3.7. Simulation and Environment

**Figure 3:**
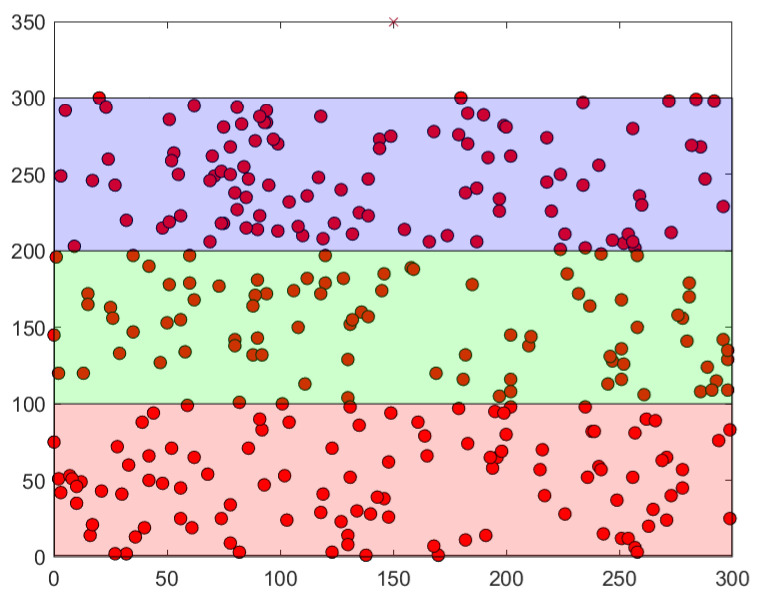
300-Node model sample.

**Figure 4:**
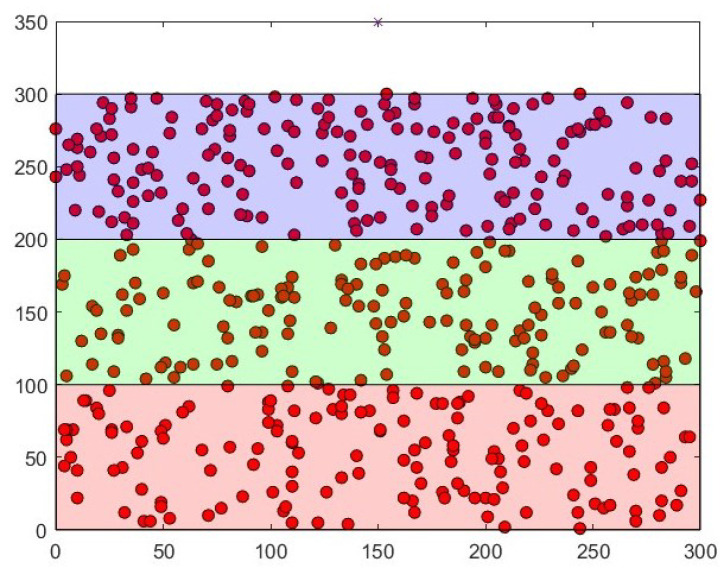
500-Node model sample.

Simulations were carried out in two phases on a DELL Latitude 55220 with 16GO RAM. The first one consisted in the evaluation of the energy consumed to implement the MAC address hashing using SHA256, as well as the encryption of messages using AES256. This part was implemented in a Tmote Sky in Contiki 3.0, an emulator for WSN and IoT where network devices are written in C. Hashing and encryption were implemented on a single node in order to calculate the energy spent for these operations. Energest is a module in Contiki that measures the number of clock ticks in different states, such as transmission, reception, CPU, sleep mode, and low power mode. During our simulations, the CPU clock ticks was the only needed value. Algorithm 1 was repeated 100 times and the average value was calculated (1078 ticks). Using the Tmote datasheet specifications, the consumed energy was obtained. The next phase consisted in simulating the SMHCA-SC. MHCA-SC was originally implemented in MATLAB. During simulations, 300, 400, and 500 nodes were randomly scattered on a 300 m × 300 m area and the sink was positioned at x = 150 m, y = 350 m during our simulations. Three 100 m wide areas were created containing nodes. Figure 3 and Figure 4 represent simulations with 300 and 500 nodes.

The message was 4000 octets in size and the energy for driving the electronics ($E_{e}lec$) was 50 nJ/bit. The coefficients $E_{f}s$ and $E_{mp}$ were respectively 10 pJ/bit/m${}^{2}$ and 0.0013 pJ/bit/m${}^{4}$. These parameters, as shown in Table 2 are used to calculate the amplifier energy $E_{amp}$. The energy consumed by hashing and encryption was introduced in the model when nodes in a same cluster send messages to their cluster head. In total, 10 randomly networks were generated for each model and the mean values were calculated.

**Figure 5:**
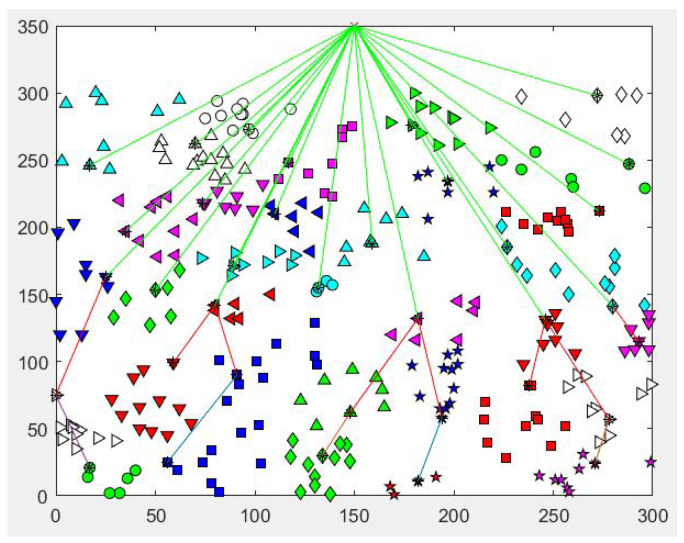
500-Node Cluster sample.

Figure 5 illustrate the resulting clusters in the network and types of communication that occur during simulations. Nodes to cluster heads communications are not shown, but CH to CH, CH to BS transmissions are observed as single hop or multi-hop messages.

## 4. Results and Discussions

### 4.1. Mean Residual Energy

**Figure 6:**
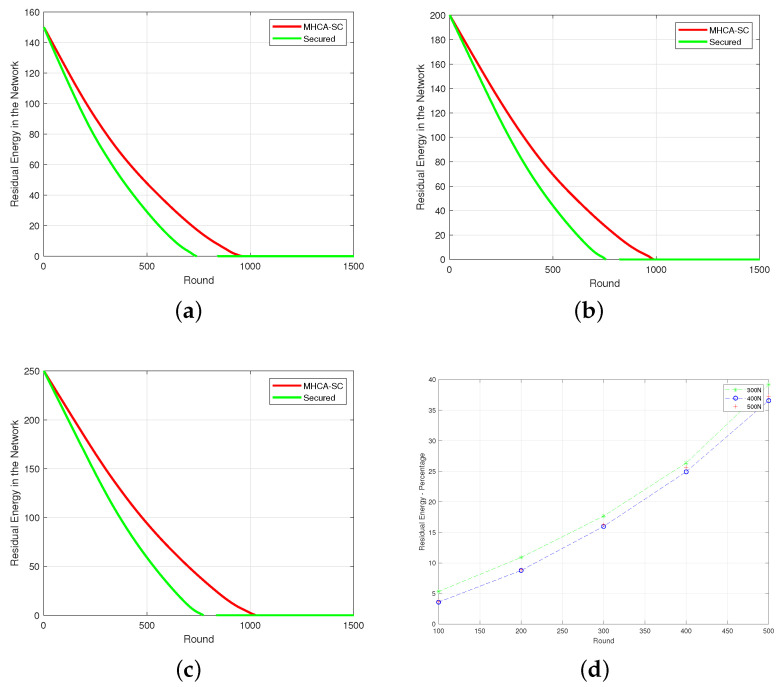
Mean residual energy. (**a**) 300-node model. (**b**) 400-node model. (**c**) 500-node model. (**d**) Residual energy percentage.

Integrating security features increased the energy spent by nodes and therefore affected the overall lifespan of the network. Figure 6a–c show that mean residual energy of SMHCA-SC remained below MHCA-SC in all scenarios (numbers of nodes). In terms of energy (Figure 6d), a 3% decrease in residual energy was observed at round 100 between the two models. The decline grew gradually to 5, 8, 16, and 37% at rounds 200, 300, 400, and 500, respectively (300/400 nodes). This means that the MHCA-SC lifespan is greater than its secure version and that security features introduce a cost in terms of residual energy. The same patter was recorded for the 500 nodes model.

**Figure 7:**
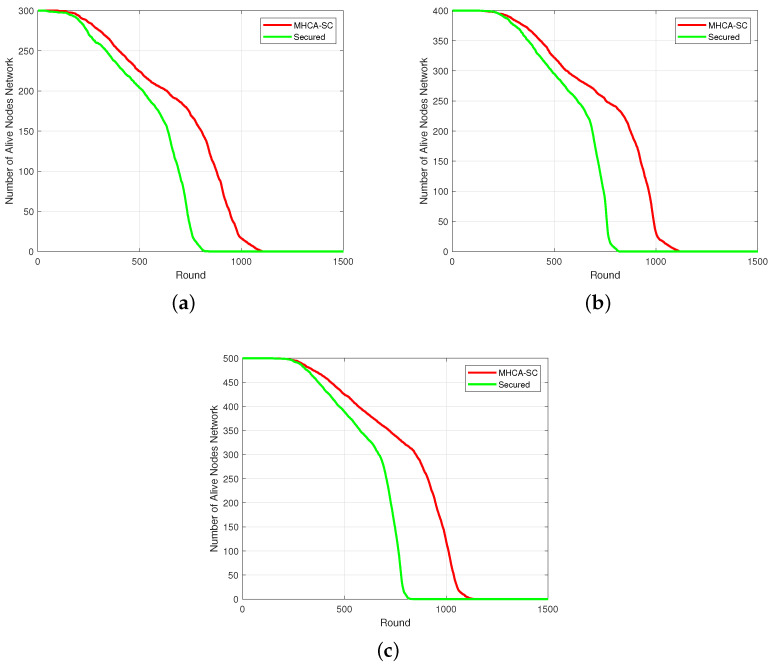
Alive nodes. (**a**) 300-node model. (**b**) 400-node model. (**c**) 500-node model.

The graph representing the number of alive nodes over time is related to the residual energy in the network. In Figure 7a, with 300 nodes at the beginning of the simulation, the curves for MHCA-SC and SMHCA-SC remained relatively close up to rounds 199 and 171, respectively, where 294 nodes were still alive. From 294 alive nodes to 150 alive nodes, the curves decline by 23% for MHCA-SC and 30% for SMHCA-SC, respectively. From 150 alive nodes, MHCA-SC curve declines 30% less than SMHCA-SC as they reached 18 alive nodes at rounds 989 and 761, respectively, and, finally, decreased by 19% and 34% before all nodes die. In Figure 7b,c, MHCA-SC decline less than SMHCA-CS, thus showing the impact of the security features on the number of alive nodes over time.

### 4.2. First Dead Node

**Figure 8:**
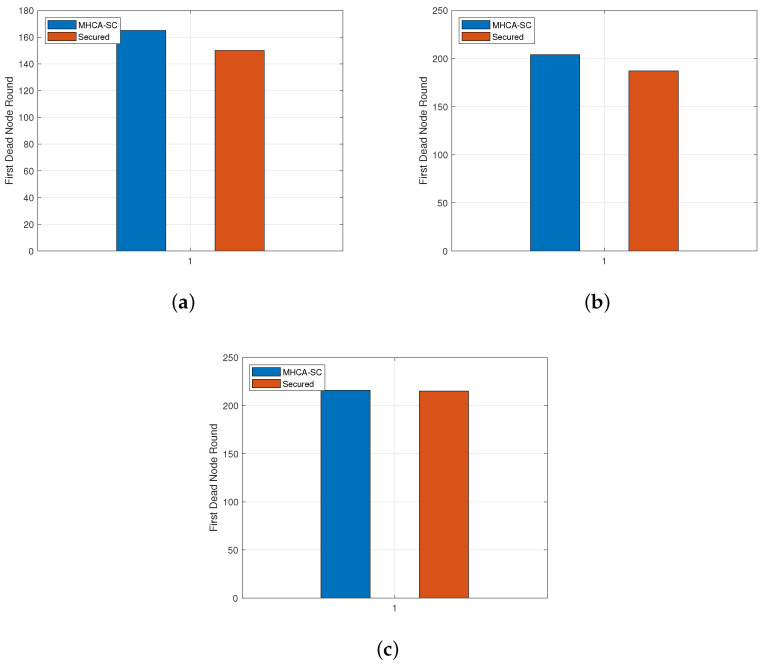
First dead node. (**a**) 300-node model. (**b**) 400-node model. (**c**) 500-node model.

Table 3 and Figure 8a–c exhibit the values obtained for the first dead node. For 300 nodes, FDN occurred at round 165 and 150 for MHCA-CS and SMHCA-CS, and at rounds 204 and 187 for 400 nodes. For 500 nodes, FDN occurred at round 216 for both protocols. Simulations with a higher number of nodes may highlight possible correlation between the number of nodes in the network and FDN occurrences.

**Figure 9:**
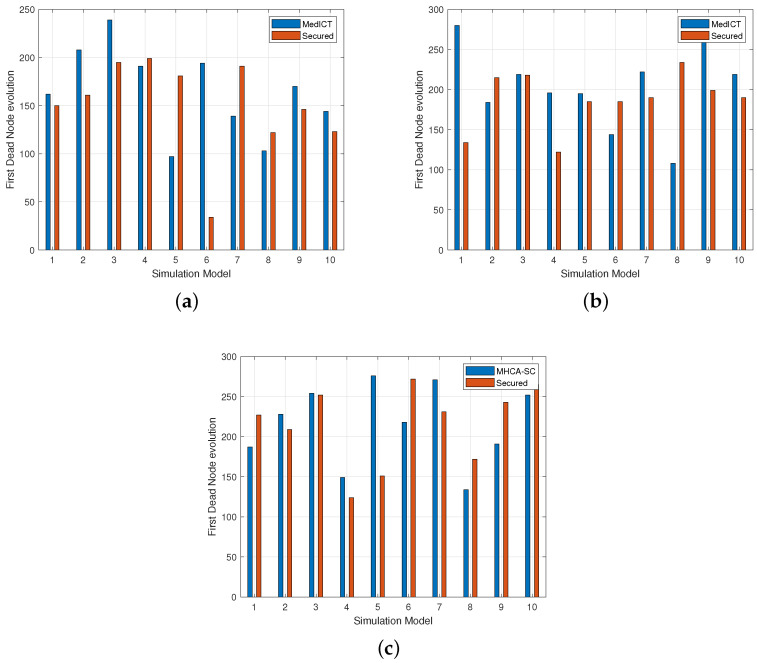
FDN evolution. (**a**) 300-node model. (**b**) 400-node model. (**c**) 500-node model.

As 10 topologies were generated, a closer look at individual simulations highlighted several cases where FDN occurred faster in MHCA-CS compared with SMHCA-CS. This was observed in simulations 4, 5, 7, and 8 of Figure 9a for the 300-node model. It also occurred in simulations 2, 6, and 8 for 400 nodes (Figure 9b), and in simulations 8 and 9 for 500 nodes (Figure 9c). These deviations could be explained by the fact that as nodes are randomly scattered in the network, for the same model simulation 5 may produce a different model for MHCA-SC and SMHCA-SC. This also suggests that other factors, such as distance and numbers of nodes in the cluster may have more impact on nodes energy and should, therefore, be thoroughly investigated.

### 4.3. Half Dead Node

As shown in Table 4, 50% of nodes died faster in SMHCA-SC compared with MHCA-SC. For 300 nodes, it was spotted at rounds 639 and 801, respectively, while the same event was observed at rounds 687 and 873 for the 400-nodes model and 707 and 910 for the 500-nodes model.

### 4.4. Last Dead Node

**Figure 10:**
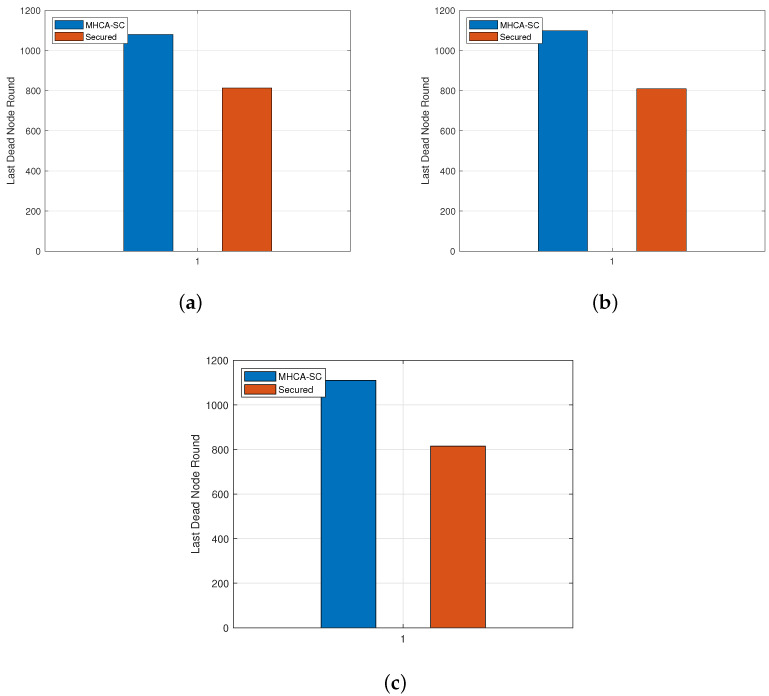
Last dead node. (**a**) 300-node model. (**b**) 400-node model. (**c**) 500-node model.

The network lifespan is also affected by cryptographic techniques. As illustrated in Table 5 for the 300-node model, all nodes were dead at rounds 1080 and 813, respectively, for MHCA-SC and SMHCA-SC. With an initial number of 400 nodes, the batteries of all sensors drained at rounds 1099 and 810 while the values were 1110 and 815 for the 500-node model. These values are shown in Figure 10.

### 4.5. LECSA vs. SMHCA-CS

**Figure 11:**
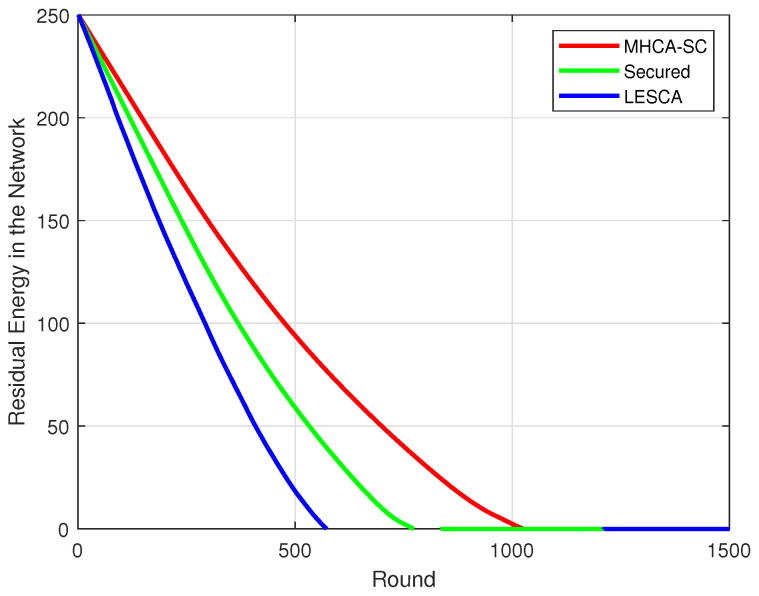
Residual energy: LESCA vs. SMHCA-CS vs. MHCA-CS (500 Nodes).

**Figure 12:**
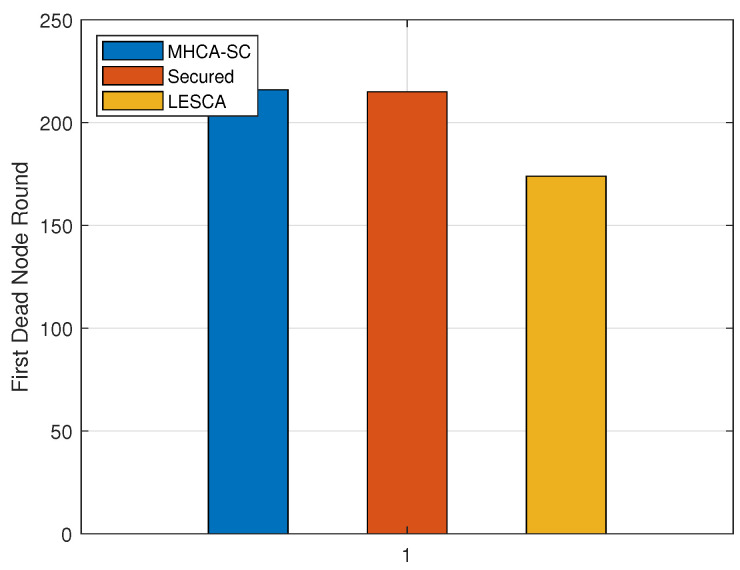
FDN: LESCA vs. SMHCA-CS vs. MHCA-CS (500 Nodes).

**Figure 13:**
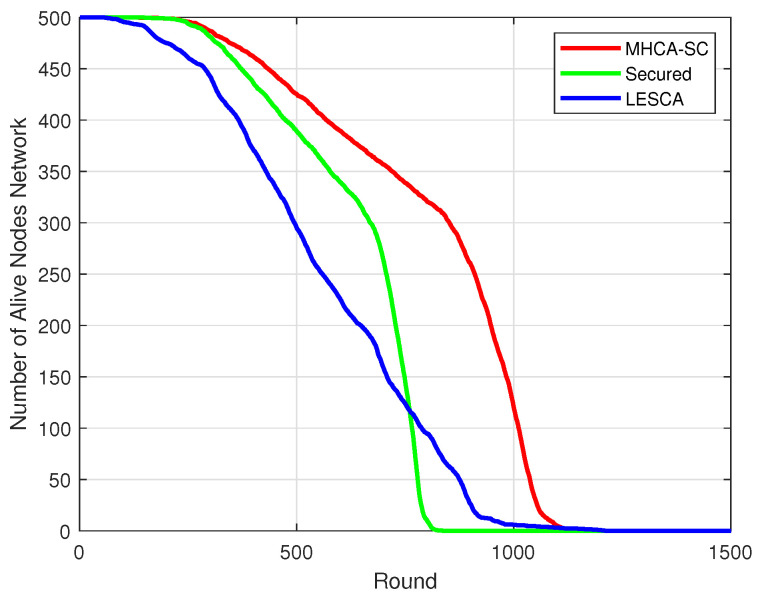
Alive nodes: LESCA vs. SMHCA-CS vs. MHCA-CS (500 nodes).

LESCA [24], a Location-Energy Spectral Clustering Algorithm, was more effective than LEACH-C and DECSA in terms of residual energy. Compared with LESCA, SMHCA-CS lasted longer in terms of mean residual energy for 500 nodes (Figure 11). FDN also occurred faster with LESCA, as shown in Figure 12. The number of alive nodes decreased at a linear rate when 90% nodes were still alive, but remained lower than SMHCA-CS up to 20% alive nodes. From 20% alive nodes, LESCA was more effective in terms of lifespan (Figure 13).

From the above results, as energy consumption was evaluated using different metrics, SMHCA-CS drained more energy in nodes compared with MHCA-CS. The number of alive nodes in SMHCA-CS declined faster compared with MHCA-CS. Half of the alive nodes and the last dead node occurred also faster in all cases. Compared with some open (not secured) cluster-based WSN, such as LESCA and SMHCA-CS, was less energy consuming. The main advantage of this work is to identify communications within cluster-based WSN and formally assess energy consumption induced to secure them. Lightweight blockchain paradigm (PoAh) ensures confidentiality, integrity, and authentication. This study’s main disadvantage is the use of two separate environments: Contiki measures energy and MATLAB evaluates the impact of security on the network related to energy. This method is not suitable to test the secured version against well known attacks such as sinkhole, wormhole, sybil attacks, and Denial Of Service because security was not implemented within MATLAB. This issue can be addressed by fully implementing a cluster-based WSN in Contiki or even NS2 for example and performing the previously mentioned attacks. It will also give an opportunity to study other metrics, such as throughput and latency, that are important in WSN.

## 5. Conclusions

Clustering usage improved WSN lifespan, load balancing, scalability, and packet delivery by rotating cluster head’s responsibilities over different nodes in clusters. CBWSN are evaluated in cluster formation, complexity, communication, and management. Security issues in WSN have been raised, especially in sensitive areas, such as military, medical, water delivery, and even smart grids, as more and more attacks surface, aiming to compromise these networks. Although some research projects propose several solutions to increase WSN safety using cryptography and ensure trustworthiness of nodes, authentication, integrity, and confidentiality, the cost in terms of energy consumption and lifespan is not formally assessed. The study formally assesses energy costs induced to secure CBWSN. The concept is applied to Multi-hop Clustering Algorithm using blockchain’s paradigm namely Power of Authentication at different levels of the communication model.

Compared with MHCA-CS, SMHCA-CS consumed more energy in general. This was precisely measured with FDN, LDN, residual energy, and the number of alive nodes. For example, with an initial number of 300 nodes, FDN was recorded at rounds 165 and 150, respectively, for MHCA-SC and SMHCA-SC. Although, for 400 nodes, FDN was spotted at rounds 204 and 187, it was noted at round 216 for MHCA-SC and SMHCA-SC. The half dead node happened at rounds 801 and 639 while LDN at rounds 1080 and 813 for 300 nodes. Compared with LESCA, SMHCA-CS exhibited better performances in terms of residual energy, FDN and the number of alive nodes at some point. For instance, the number of alive nodes decreased at a linear rate when 90% of nodes were alive but remained below SMHCA-CS.This illustrates the need for lightweight security algorithms. Although this study focuses on Multi-hop Clustering Algorithm based on spectral classification, it was implemented on two different environments, thus preventing simulations of well-known attacks that may occur in a WSN. Future works include a full implementation of a cluster-based WSN and its secured version, its robustness against several know attacks, as well as the evaluation of other metrics.

## Figures and Tables

**Table 1:** Extra consumed energy evaluation to secure MHA-CS.

Node to CH	CH to CH	CH to CH	CH to BS	Final	Consumed Energy
**C1**	**C1 to C2**	**C2 to C3**	**C3 to BS**		
(1)	(2)	(4)	(6)	(1) (2) (4) (6)	$E_{1}=\left(1\right)+\left(2\right)+\left(4\right)+\left(6\right)$
			(7)	(1) (2) (4) (7)	$E_{2}=\left(1\right)+\left(2\right)+\left(4\right)$
		(5)	(6)	(1) (2) (5) (6)	$E_{3}=\left(1\right)+\left(2\right)+\left(6\right)$
			(7)	(1) (2) (5) (7)	$E_{4}=\left(1\right)+\left(2\right)$
	(3)	(4)	(6)	(1) (3) (4) (6)	$E_{5}=\left(1\right)+\left(4\right)+\left(6\right)$
			(7)	(1) (3) (4) (7)	$E_{6}=\left(1\right)+\left(4\right)$
		(5)	(6)	(1) (3) (5) (6)	$E_{7}=\left(1\right)+\left(6\right)$
			(7)	(1) (3) (5) (7)	$E_{8}=\left(1\right)$

**Table 2:** Simulation parameters.

Parameter	Value
$E_{elec}$	50 nJ/bit
$E_{fs}$	10 pJ/bit/m${}^{2}$
$E_{mp}$	0.0013 pJ/bit/m${}^{4}$
$E_{DA}$	5 nJ/bit/message
$d_{0}$	88 m
Message Size	4000 bytes
Area	300 m × 300 m
Zone 1	(x∈[0,300], y∈[0,100])
Zone 2	(x∈[0,300], y∈[100,200])
Zone 3	(x∈[0,300], y∈[200,300])
Sink Position	(150 m, 350 m)
Number of Nodes	300, 400, 500

**Table 3:** First dead node.

	300 N	400 N	500 N
MHCA-SC	165	204	216
SECURED MHCA-SC	150	187	216

**Table 4:** Half dead node.

	300 N	400 N	500 N
MHCA-SC	801	873	910
SECURED MHCA-SC	639	687	707

**Table 5:** Last dead node.

	300 N	400 N	500 N
MHCA-SC	1080	1099	1110
SECURED MHCA-SC	813	810	815

## Sample Availability

Data are available on request to authors.

## Abbreviations

The following abbreviations are used in this manuscript:

AES	Advanced Encryption Standard
BS	Base Station
CPU	Central Processing Unit
CBWSN	Cluster-based Wireless Sensor Network
CH	Cluster Head
CM	Cluster Member
DECSA	Distance-Energy Cluster Structure Algorithm
DOS	Denial Of Service
EACLE	Energy-Aware Clustering Scheme with Transmission Power Control for Sensor Networks
EATSRA	Energy Aware Trust-based Secured Routing Algorithm
EAFCA	Energy Aware Fuzzy Clustering Algorithm
ECPF	Energy-aware Distributed Dynamic Clustering Protocol using Fuzzy Logic
EEHC	Energy Efficient Heterogeneous Clustered scheme
EERC	Energy Efficient Recursive Clustering
FDN	First Dead Node
HCA-SC	Hierarchical Clustering Algorithm Based on Spectral Classification
HEED	Hybrid Energy Efficient Distributed clustering
LBSWSN	Lightweight Blockchain for Secure Wireless Sensor Network
LDN	Last Dead Node
LEACH	Low Energy Adaptive Clustering Hierarchy
LEACH-C	Low Energy Adaptive Clustering Hierarchy Centralized
LESCA	Location-Energy Spectral Clustering Algorithm
MAC	Media Access Control
MHCA-SC	Multi-hop Clustering Algorithm based on Spectral Classification
POAh	Proof-Of-Authentication
QOS	Quality Of Service
RAM	Random Access Memory
RSS	Received Signal Strength
SEDEEC	Stochastic and Equitable Distributed Energy-Efficient Clustering
SCNOC	Spectral Classification based on Near Optimal Clustering
SHA	Secure Hash Algorithm
SMHCA-SC	Secured Multi-hop Clustering Algorithm based on Spectral Classification
TDMA	Time Division Multiple Access
WSN	Wireless Sensor Network
